# Softening of the Brain

**Published:** 1877-09-20

**Authors:** 


Softening of the Brain.—Dr. W. W. Parker, before the Richmond Academy of Medicine, stated " that this condition often exists for long periods without being suspected." He knew of a man whose mental faculties were unimpaired twenty-four hours before death, yet post-mortem examination showed it to be very soft. He also had a similar experience in a child, "in whom the condition was not suspected until shortly before death. Dr. L. B. Edwards calls softening a necrosis, and thinks it sometimes the result of a primary breaking down of brain cells, not always due to occluded vessels. He believes "nerve cells have an inherent instability not solely dependent on the immediate blood supply. He thinks phosphorus and strychnia, in combination, are the best tonic agents. He also endeavors to keep the "necrosis" from "extending beyond the original locality" by efforts to establish collateral circulation, so that the parts beyond need not suffer for lack of nourishment. I suppose this has reference to the common cases in which there is occulsion of some vessel. The Doctor advised the judicious use of nitrite of amyl, long continued, and three or four times a day, to meet the above indication.

